# Incentive-Based Telematics and Driver Safety: Insights from a Naturalistic Study of Behavioral Change

**DOI:** 10.3390/s25247433

**Published:** 2025-12-06

**Authors:** Armira Kontaxi, Haris Sideris, Dimitris Oikonomopoulos, George Yannis

**Affiliations:** 1 Department of Transportation Planning and Engineering, National Technical University of Athens, GR-15773 Athens, Greece; geyannis@central.ntua.gr; 2 OSeven Single Member Private Company, GR-15234 Athens, Greece; hsideris@oseven.io (H.S.); doikonomopoulos@oseven.io (D.O.)

**Keywords:** road safety, driver profiling, telematics, gamification, incentive-based intervention, smartphone sensors, K-means clustering, naturalistic driving

## Abstract

**Highlights:**

**What are the main findings?**

- Three distinct driver profiles were identified through clustering of smartphone-based telematics data: Low-Exposure Cautious, Balanced/Average, and High-Risk Drivers.
- Balanced/Average and High-Risk Drivers showed statistically significant reductions in speeding during incentive-based challenges, confirming the positive impact of gamified feedback schemes.

**What is the implication of the main finding?**

- Incentive-based telematics interventions can effectively promote safer driving behavior, particularly among moderately risky drivers.
- Driver profiling enables the design of personalized feedback and incentive mechanisms for usage-based insurance and road safety programs.

**Abstract:**

Understanding how drivers respond to feedback and incentives is crucial for designing data-driven interventions that enhance road safety. This study investigates driver profiling and behavioral change using high-resolution telematics data collected through the OSeven DrivingStar smartphone application within the O7Insurance project. The naturalistic driving experiment was divided into two main phases: a baseline period with personalized feedback (Phase A) and an incentive-based phase (Phase B) comprising two gamified driving challenges with distinct reward criteria. Using data from 86 active participants, K-means clustering identified three driver profiles—Low-Exposure Cautious, Balanced/Average, and High-Risk Drivers—based on exposure, harsh events, speeding, and mobile phone use. The Balanced/Average group exhibited statistically significant improvements during both challenges, reducing speeding frequency and intensity (e.g., from 4.8% to 3.7%, *p* < 0.01), while High-Risk Drivers achieved moderate reductions in speeding intensity (from 6.4 to 5.3 km/h, *p* < 0.05). Low-Exposure Cautious Drivers maintained stable, low-risk performance throughout. These findings demonstrate that incentive-based telematics schemes can effectively influence driving behavior, particularly among drivers with moderate risk levels. The study contributes to the growing body of research on gamified driver feedback by linking behavioral clustering with responsiveness to incentives, providing a foundation for adaptive and personalized road safety interventions.

## 1. Introduction

Despite notable advancements in road safety over the past decade, road traffic crashes continue to represent a major global public health concern, causing approximately 1.19 million deaths in 2021, equivalent to a rate of 15 fatalities per 100,000 population [1]. Extensive research has sought to identify the key risk factors contributing to road traffic crashes, with human-related factors consistently emerging as the most influential. Indeed, human error has been attributed to about 95% of all crashes [2]. This highlights the critical role of understanding and addressing driver behavior as a cornerstone of road safety strategies. Through the systematic analysis of driving behavior, it becomes possible to design targeted interventions aimed at reducing risky actions such as distraction, speeding, and impaired driving.

In that framework, the Internet of Things (IoT) constantly offers new opportunities and features to monitor and analyze driver behavior through wide use of smartphones, effective data collection and Big Data analysis, resulting in assessment and improvement of driver behavior and safety [3,4]. More precisely, technological advancements in driver behavior telematics are exploited by the insurance industry, which has been growing and changing rapidly over the last two decades. In addition, the high penetration rate of smartphones has offered new possibilities for faster, more accurate, and low-priced driver behavior data collection [5,6]. The interpretation of this data is possible thanks to progress in computing power, data science and artificial intelligence.

Previous studies have concluded with several interesting and useful results regarding the effect of various types of telematics on driving performance and safety. More specifically, recent review studies have examined the utility of telematics data for road safety, showcasing promising results with respect to crash risk reduction and improvement of driving behavior [7,8,9,10]. It is also highlighted that personalized target setting, benchmarking and comparison with peers may have a greater impact [11,12,13].

For example, a recent systematic review [14] examined naturalistic driving studies investigating the impact of driver feedback on driving behavior and road safety. Drawing on 34 studies identified through a PRISMA-based framework, it found that feedback interventions, delivered via smartphone applications, in-vehicle systems, and web-based platforms, consistently reduced risky behaviors such as speeding and harsh braking, thereby improving safety outcomes, while also emphasizing the need for further research on long-term effectiveness, contextual factors, and the integration of emerging technologies such as IoT and machine learning.

Driver profiling through incentive-based cluster analysis offers a powerful approach to understanding heterogeneous driving behavior in real-world conditions. By leveraging data collected from naturalistic driving studies, such as telematics-based trip records, researchers can apply unsupervised learning techniques like K-means or hierarchical clustering to group drivers based on behavioral indicators [15,16]. For instance, a recent study [17] employed a two-level k means clustering approach using smartphone-based sensor data to distinguish between aggressive, distracted, and safe driving styles. By analyzing variables such as harsh events, acceleration profiles, mobile phone use, and speeding, six distinct trip categories were identified, revealing that nearly half of all trips were safe while a significant share involved speeding or distraction.

Overall, these profiles are often used to identify risk-prone or eco-conscious driver types. Specifically, one study [18] developed a comprehensive risk assessment model using lateral and longitudinal driving indicators, such as stability, car-following, and lane-changing risk, to classify drivers into four behavioral categories: dangerous, aggressive, safe, and conservative. By combining statistical trajectory analysis and K-means clustering, the study demonstrated that multi-criteria behavioral indicators can reliably identify real-time driving risks and support proactive safety warning systems. Another study [19] applied K-means cluster analysis to self-reported measures of driving behavior (DBQ) and driving skill (DSI) and identified five clusters: namely, unsafe and offensive, safe and skillful, unsafe and relatively unskilled, unskilled and relatively unsafe, and safe drivers with low self-confidence. Results revealed that lower self-reported skill levels were generally associated with higher frequencies of aberrant driving behaviors.

Furthermore, other studies [20,21] highlight the utility of clustering in usage-based insurance (UBI) and personalized interventions. However, there is still limited research that examines these driver profiles within the framework of incentive schemes, such as competitions or gamified challenges, to assess not only how individuals drive but also how they respond to behavioral nudges.

In light of the aforementioned, the objective of the present study is twofold: (a) to conduct a profiling of drivers through cluster analysis to better understand behavior patterns, and (b) to examine the impact of personalized challenges and incentives on driver behavior. Specifically, to clarify the research novelty and contribution of this study, the following points summarize the key innovations and advancements introduced by our work:

- The study introduces an integrated framework that combines real-world telematics data, gamification-based incentives, and cluster analysis to investigate driver behavior.
- It moves beyond traditional approaches that examine gamification or telematics in isolation or within simulated environments.
- It applies unsupervised clustering to identify distinct driver profiles, revealing heterogeneous behavioral responses to incentive-based interventions.
- It contributes to the understanding of how personalized and data-driven feedback can enhance engagement, improve safety performance, and support long-term behavioral change.
- The approach provides actionable insights into fleet management, usage-based insurance, and mobility behavior research

The study is structured as follows: Section 2 presents the materials and methods, including the experimental design, smartphone application, and theoretical background of the analytical methods employed. Section 3 discusses the results of the analysis, focusing on both driver profiling and the effects of the incentive-based challenges on driving behavior. Section 4 provides a detailed discussion of the key findings, linking them with previous research, identifying limitations, and outlining directions for future work. Finally, Section 5 summarizes the main conclusions of the study.

## 2. Materials and Methods

### 2.1. The DrivingStar Application

**Figure 1:**
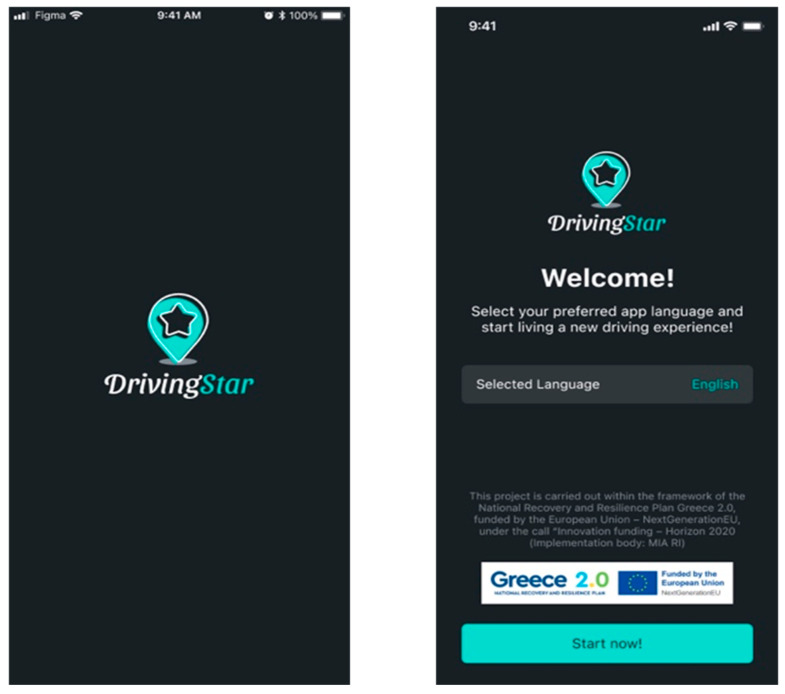
Screenshots of the DrivingStar smartphone application.

In order to achieve the research objective, an innovative smartphone application developed by OSeven (www.oseven.io) (Athens, Greece) for the purpose of the “O7Insurance” (implemented under the National Recovery and Resilience Plan Greece 2.0) research project was exploited, aiming to record, analyze and improve driver behavior. “O7Insurance” introduces a novel approach to vehicle insurance management by enabling drivers to handle all aspects of their coverage through a mobile application, DrivingStar, supported by OSeven’s telematics technology. By continuously rewarding safe and eco-friendly driving behavior with monetary incentives, the system is designed to promote improved driving habits, leading to lower premiums, fewer claims, and a reduction in both road accidents and environmental impact. A screenshot of the DrivingStar smartphone application is presented in Figure 1.

**Figure 2:**
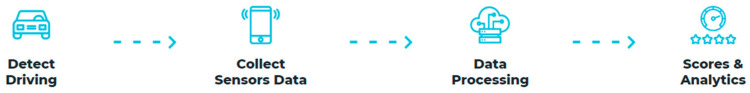
The OSeven data flow system.

Within the framework of the “O7Insurance” project, OSeven has also developed a seamless integration platform for collecting and transferring raw data and recognizing the driving behavior metrics via Machine Learning (ML) algorithms. After the end of each trip, the application transmits all data recorded to the central database of the OSeven backend platform via an appropriate communication channel, such as a Wi-Fi network or cellular network (upon user’s selection), e.g., 3G/4G (online options). The data collected is highly disaggregated in terms of space and time. Also, the data provided by OSeven for the current analysis was in a completely anonymized format, in full compliance with GDPR and personal data protection policies the company has in place. The standard procedure that is followed every time a new trip is recorded by the application is shown in Figure 2.

A variety of different metadata are eventually calculated, including the following exposure indicators:

- Total distance (mileage)
- Driving duration
- Type(s) of the road network used (given by GPS position and integration with map providers, e.g., Google, OSM)
- Time of the day driving (rush hours, risky hours)
- Weather conditions (under development, on the basis of integration with weather data providers)
- Trip characterization (predicted by sensor data and confirmed or rejected by the user, whether the user was driving or was using other modes of transport)

The driving behavior indicators that are also calculated from the data include:

- Speeding (duration of speeding, speed limit exceedance, etc.)
- Number and severity of harsh events
- Harsh braking (longitudinal acceleration)
- Harsh acceleration (longitudinal acceleration)
- Distraction from mobile phone use (mobile phone use is considered to be any type of phone use by the driver, e.g., talking, texting, etc.)
- Eco Driving Indicator

**Figure 3:**
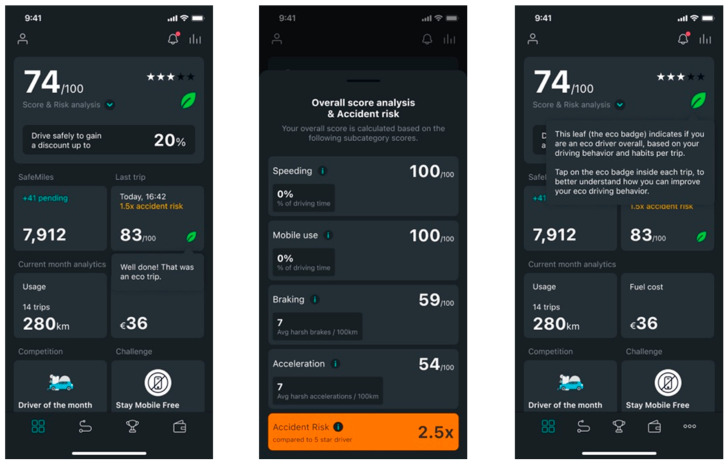
Screenshots of the DrivingStar smartphone application, illustrating the app’s indicators.

Figure 3 illustrates the application’s indicators.

In the context of the above indicators, the following methodological clarifications are provided regarding the detection and processing of speeding, harsh events, and mobile phone use. Harsh events (e.g., harsh braking and harsh acceleration) are determined through OSeven’s proprietary algorithms, which employ data fusion and machine learning techniques combining data from multiple smartphone sensors (accelerometer, gyro-scope, magnetometer, and GPS) to identify event patterns rather than relying on fixed thresholds. The minimum sampling frequency of the collected signals is typically 1 Hz, while for certain sensors, the effective sampling frequency may be higher. Although the exact parameter values cannot be disclosed due to intellectual property protection, these algorithms have been extensively validated against on-board diagnostics (OBD) data, expert on-road assessments, and driving simulator experiments [5,22], and are already implemented by major insurance companies, confirming their reliability and accuracy.

As for the speeding percentage, the variable refers to instances when drivers exceed the posted speed limits on the respective road network, identified through comparison of the treated (smoothed and outlier-filtered) GPS speed values recorded by the DrivingStar application with the corresponding OpenStreetMap limits. A small tolerance margin is applied to each speed limit to account for potential GPS deviations. In addition, the detection of mobile phone use (talking, texting, or app interaction) relies exclusively on smartphone sensors, flagging each second of use while the vehicle is moving, with automated recognition between driving and stationary states (accuracy approximately 95%) and post-trip user confirmation to ensure fair and accurate distraction assessment.

### 2.2. Experimental Design

For the purposes of the O7Insurance project, OSeven conducted a pilot program that ran from March 2024 to March 2025. The aim of this pilot was to test and optimize the functionality of the O7Insurance vision in real-world conditions, introducing a new era in vehicle insurance management by enabling drivers to handle all aspects of their coverage through a mobile application. More than 320 drivers registered with the DrivingStar app, collectively recording approximately 70,000 trips and 600,000 km on roads in Greece and abroad.

**Figure 4:**
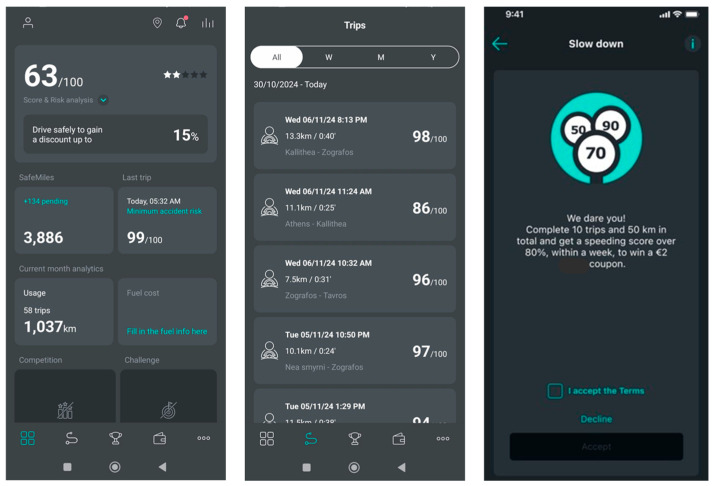
Example screenshots from the application features in Phase A—Baseline (**left**,**middle**) and Phase B—Challenges (**right**).

The naturalistic driving experiment was structured into two main phases: Phase A (Baseline) and Phase B (Challenges), which differed in the type of feedback and incentives provided to drivers, as shown in Figure 4. Phase A served as the baseline period (1 September 2024–4 December 2024), during which participants received only personalized feedback through the mobile application, including scorecards, statistics, and reports on their driving behavior. This phase allowed the assessment of natural driving patterns before the introduction of any incentive-based interventions.

In Phase B, two separate gamified challenges were implemented, each with distinct characteristics and reward criteria, aiming to evaluate the effectiveness of incentives on improving driving behavior (Table 1). The first challenge lasted 23 days (5–27 December 2024), during which drivers who completed at least 10 trips and 50 km while maintaining a speeding score above 80/100 within a week were eligible for recognition and small-value (2 €) coupons redeemable through a mobile delivery app. The second challenge, conducted between 30 January and 10 February 2025, required invited drivers to complete at least 5 trips and 40 km while achieving an overall driving score above 75/100, rewarding them with 5 € fuel-discount coupons.

More precisely, the rewards provided are explained below:

- The digital vouchers used in the first challenge were 2 € incentives redeemable through a Greek food-delivery app.
- The fuel-discount vouchers used in the second challenge were 5 € credits redeemable at participating fuel stations of a major fuel provider in Greece.

The two challenges differed in duration due to their behavioural focus. Challenge 1 targeted a single performance indicator (speeding), requiring additional time for drivers to complete the minimum number of trips and to demonstrate change in that specific metric. Challenge 2 was based on the overall safety score, an aggregate indicator for which the minimum trip requirement was sufficient to produce stable measurements, allowing for a shorter challenge window.

For the purpose of the present analysis, a subtotal of 86 drivers who actively participated in both challenge phases were retained to evaluate the effect of incentive-based interventions on driving behavior over the period 1 September 2024–10 February 2025, when the second challenge concluded.

Furthermore, it should be noted that all data processing complies with the EU GDPR under OSeven. Users provide explicit consent to the Terms of Use and Privacy Notice upon app installation and registration. Personal identifiers (e.g., email or account details) are stored separately from driving behaviour data, which are linked only through randomly generated unique IDs. This ensures that individual users cannot be directly identified during analysis. All data are stored on secure EU-based servers, processed solely for the purposes defined in the Privacy Policy, and protected through encryption and strict access controls.

### 2.3. Theoretical Background

#### 2.3.1. K-Means Clustering

Clustering is useful in order to divide the driver sample into several categories, which can provide insights as to whether driving behavior differs systematically on a macroscopic scale. K-means clustering is a very well-known and straightforward algorithm used to separate datasets into clusters, and belongs to the unsupervised machine learning algorithms. The algorithm searches for a specific number (*k*) of clusters in a dataset. The algorithm first initiates by randomly selecting centroids in the data. Each data point is then assigned to the nearest centroid, forming *k* clusters. Centroids are recomputed for the formed clusters, and thus their location changes. Calculations are then performed to re-assign each data point to its new centroid. Afterwards, iterative calculations are conducted until no reassignments are made and thus the centroids have stabilized.

The popularity of K-means algorithms presented in the past (e.g., [22]) has led to several customized approaches in the literature (e.g., [23,24]). K-means has been used widely for clustering purposes in several transport/road safety studies as well (e.g., [17,25]).

There are a number of methods to determine the optimal number of clusters for any dataset; in the current study, the elbow method is followed, which is used to determine the optimal number of clusters by identifying the point where the within-cluster sum of squared errors begins to decrease at a decreasing rate [26].

#### 2.3.2. Wilcoxon Signed-Rank Test

The Wilcoxon Signed-Rank Test is a non-parametric alternative to the Paired Samples *t*-test, used when the data do not meet the normality assumption. This test compares the median of paired differences and is robust to outliers and skewed distributions. For instance, it can be used to assess whether a mindfulness program affects stress levels, where pre- and post-program stress scores are ranked and analyzed.

The procedure involves ranking the absolute differences between paired observations, assigning positive or negative signs based on the direction of the difference, and calculating the test statistic, which is the sum of signed ranks:

(1) $$W=\sum \;R_{i}^{+}$$

where $R_{i}^{+}$ are the ranks with positive differences.

The test statistics are then compared to a critical value from the Wilcoxon Signed-Rank distribution or converted into a z-score for large samples:

(2) $$z=\frac{W-\mu_{W}}{\sigma_{W}}$$

where $\mu_{W}$ and $\sigma_{W}$ are the mean and standard deviation of the rank sum distribution under the null hypothesis. Like the *t*-test, if the *p*-value is below the significance level (α), the null hypothesis (H0: no difference in medians) is rejected. Both tests provide valuable insights into paired data, with the Wilcoxon Signed-Rank Test offering a more robust solution when the normality assumption is violated.

## 3. Results

### 3.1. Data Processing

Regarding the data processing method, all necessary data preparation steps were conducted during the pre-analysis phase to ensure quality and consistency. Outlier values in continuous indicators (e.g., speeding intensity, harsh event frequencies) were identified using standard statistical criteria based on the interquartile range (IQR) method and excluded when exceeding 1.5 × IQR above the third quartile, as extreme upper values were rare and could disproportionately influence the results. Trips containing missing or incomplete sensor data were removed to maintain uniformity across indicators. Furthermore, all continuous behavioral variables were standardized using z-score normalization before applying the K-means clustering algorithm, ensuring that differences in measurement scale did not bias the results. Finally, the final selection of variables reflects key exposure and behavioral factors that are strongly linked to driving risk and safety performance. The description and corresponding units of both exposure and behavioral variables used in the analysis are presented in Table 2.

### 3.2. Driver Profiling

**Figure 5:**
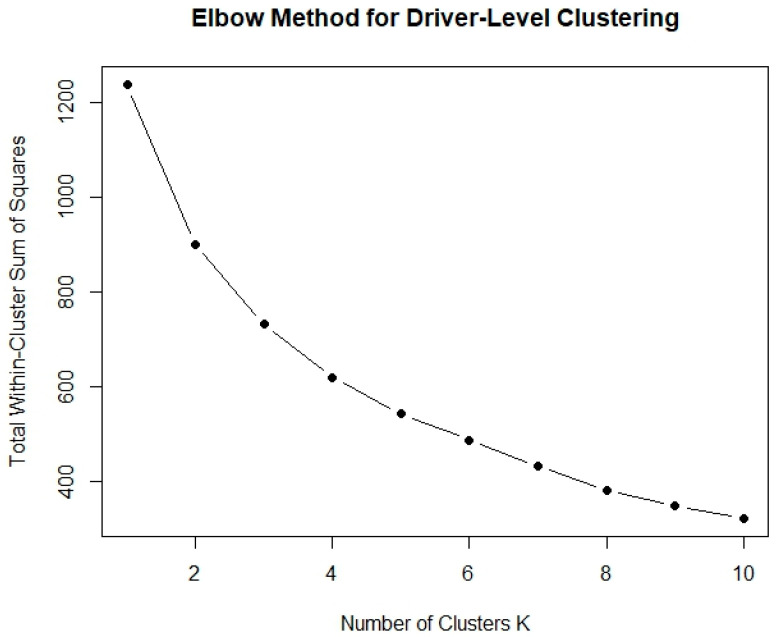
Elbow Method for cluster number determination.

After establishing the theoretical background, several clustering configurations were tested, utilizing the algorithm of [22] for K-means clustering (default for R-Studio). Based on the elbow method presented in Figure 5, the optimal number of clusters for driver-level behavior segmentation appears to be three, as the within-cluster sum of squares begins to level off, indicating diminishing returns from additional clusters. Using this approach, the K-means algorithm identified three distinct clusters that correspond to meaningful driver profiles. For the purposes of this analysis, only data from the baseline phase (Phase A) were utilized to develop these profiles, which subsequently served as the foundation for examining the effects of the incentive-based challenges in Phase B.

**Figure 6:**
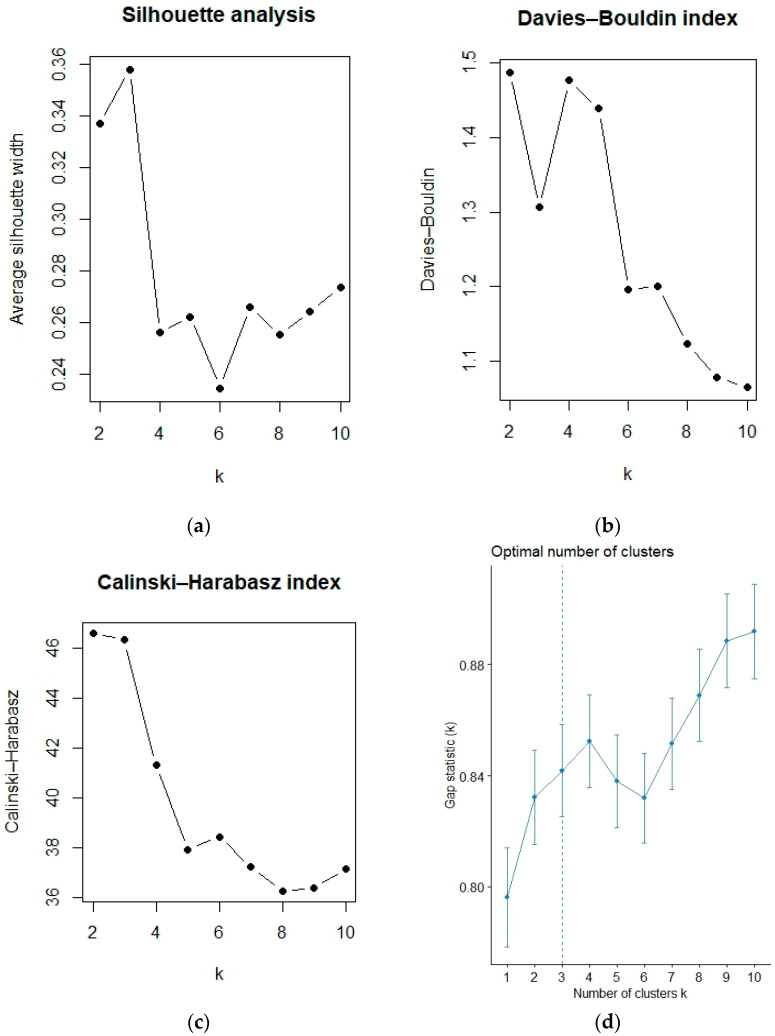
Optimal number of clusters based on multiple validity indices: (**a**) Silhouette coefficient, (**b**) Davies–Bouldin index, (**c**) Calinski–Harabasz index, and (**d**) Gap statistic.

To validate the optimal number of clusters, additional clustering validity indicators were examined beyond the elbow method. Specifically, the silhouette coefficient, the Calinski–Harabasz index, the Davies–Bouldin index, and the Gap statistic were computed for different values of k (from 2 to 10). As illustrated in Figure 6 the silhouette and Calinski–Harabasz indices reached their local maxima at k = 3, while the Davies–Bouldin index reached a corresponding local minimum, and the Gap statistic also indicated k = 3 as the optimal solution. These results jointly confirm that the three-cluster configuration achieves a satisfactory balance between within-cluster homogeneity and between-cluster separation, thus validating the robustness of the selected driver profiles.

**Figure 7:**
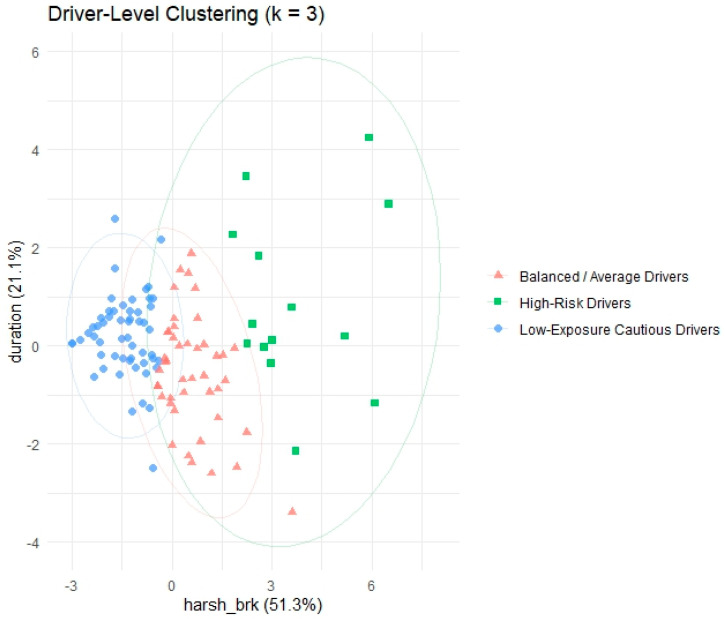
**Two-dimensional** Cluster visualization.

Table 3 and Figure 7 collectively characterize these clusters. The Low-Exposure Cautious Drivers group includes drivers with the lowest total distance and duration, suggesting infrequent driving. They also show the lowest levels of harsh events (acceleration and braking), speeding, and moderate mobile phone use—indicative of conservative, safe behavior. The Balanced/Average Drivers cluster covers those with moderate travel distance and duration, slightly elevated harshness and speeding indicators, and the lowest mobile phone usage, suggesting relatively stable yet active drivers. Finally, the High-Risk Drivers cluster is distinguished by the highest values in nearly all risk-related variables: extreme harsh events, significant speeding both in frequency and magnitude, and elevated mobile phone use during trips. These drivers also had the longest driving durations, indicating not only risky behavior but also high exposure on the road.

This clustering outcome highlights clear behavioral distinctions that can inform tailored interventions. For example, cautious drivers might be maintained with light feedback, while high-risk drivers could be targeted for more intensive coaching, incentives, or restrictions. Additionally, the profile-based segmentation enables more nuanced evaluation of future campaigns or policy interventions aimed at improving road safety.

### 3.3. Impact of Challenges on Driving Behavior

This subsection investigates the impact of incentive-based driving challenges implemented during the experiment, aiming to assess whether such interventions led to measurable improvements in key driving behavior indicators across distinct driver profiles. Mean values and Wilcoxon Test results for driving behavior indicators before and during the 1st and the 2nd challenge, accordingly. Given the number of comparisons across profiles, challenges, and behavioral indicators, the statistical results should be interpreted as exploratory. Accordingly, we focus on the direction and magnitude of changes rather than on *p*-value thresholds. The Wilcoxon signed-rank test does not always provide confidence intervals when paired differences are tied or zero; in such cases, we report the median changes descriptively. This framing aims to emphasize practical significance and behavioral relevance over binary statistical significance.

#### 3.3.1. First Challenge

**Figure 8:**
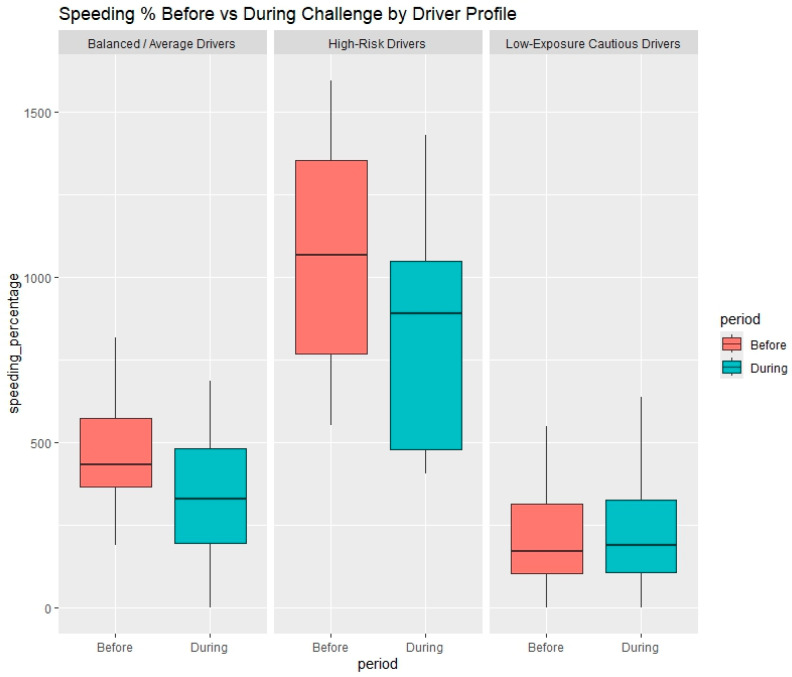
Speeding percentage before and during the 1st challenge period by driver profile.

The results from challenge 1, shown in Table 4 and Figure 8, reveal how different driver profiles responded to the safe driving challenge in terms of speeding behavior. A total of 63 drivers participated in this challenge, and their performance is compared against their respective baseline driving behavior. Findings show that the Balanced/Average Drivers exhibited a small but statistically significant reduction in both speeding percentage (from 4.6% to 4.4%, *p* = 0.04) and average speeding intensity (from 3.8 to 3.5 km/h, *p* = 0.03). This suggests that this group was moderately responsive to the incentive mechanism of the challenge, potentially due to their moderate baseline risk and room for improvement.

In contrast, High-Risk Drivers, although showing a noticeable numerical drop in speeding percentage and mobile phone use, did not exhibit statistically significant changes (*p* > 0.05 for all variables except speeding_kmh_avg). Their average speed decreased from 6.1 to 5.7 km/h, which was statistically significant (*p* = 0.03), indicating a partial effect of the intervention. However, their overall speeding behavior remained the highest across all groups, suggesting that high-risk drivers may require more intensive or personalized interventions to meaningfully alter risky habits.

The Low-Exposure Cautious Drivers showed the least variation in their behavior before and during the challenge, with no statistically significant differences across any variables. This stability is consistent with their baseline profile: low driving exposure, low harsh events, and minimal risky behavior. Their limited room for improvement also implies that challenges may have less influence on this group, making them ideal candidates for ongoing low-touch reinforcement rather than behavior-change campaigns.

#### 3.3.2. Second Challenge

**Figure 9:**
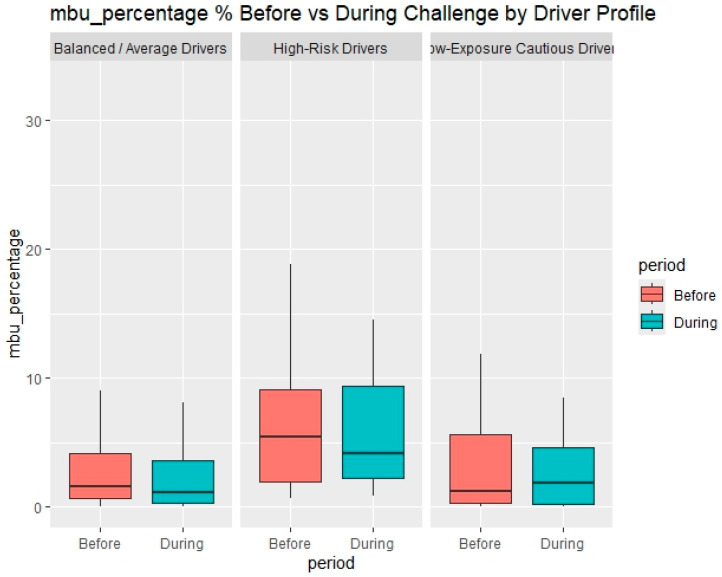
Mobile use percentage before and during the challenge period by driver profile.

The results from Challenge 2, presented in Table 5 and Figure 9, illustrate the effects of the second incentive-based intervention on different driver profiles. A total of 61 drivers participated in this challenge, and their performance is compared against their respective baseline driving behavior. Overall, the findings indicate that the introduction of a shorter challenge period with modified reward criteria led to more pronounced behavioral improvements among both Balanced/Average Drivers and High-Risk Drivers, particularly regarding speeding indicators.

The Balanced/Average Drivers showed statistically significant reductions in all speeding-related measures. Their average speeding percentage decreased from 4.80% to 3.67% (*p* = 0.001), and their average speeding intensity dropped from 3.84 to 3.08 km/h (*p* = 0.002). Additionally, a modest but significant decrease in mobile phone use was observed (from 2.90% to 2.46%, *p* = 0.048). These results confirm that this group continued to respond positively to gamified incentives, possibly reflecting their moderate baseline risk and receptiveness to feedback-based motivation mechanisms.

The High-Risk Drivers also exhibited significant behavioral improvements, especially regarding speeding. Their speeding percentage decreased from 10.66% to 8.47% (*p* = 0.018), and their average speeding intensity declined from 6.40 to 5.33 km/h (*p* = 0.045). Although reductions in harsh events and mobile phone use were not statistically significant (*p* > 0.05), the overall downward trend suggests that even high-risk drivers may benefit from repeated exposure to incentive-based interventions, albeit at a slower rate of behavioral adaptation.

In contrast, the Low-Exposure Cautious Drivers showed minimal variation in their driving behavior before and during the second challenge. None of the changes observed across harsh events, speeding, or mobile phone use reached statistical significance (*p* > 0.05). This stability is consistent with their low-risk profile and limited exposure, leaving less room for measurable improvement.

Taken together, these results indicate that the second challenge reinforced the findings from the first intervention. Incentive-based schemes appear particularly effective for Balanced/Average Drivers, who demonstrate both moderate risk and adaptability, while High-Risk Drivers show partial responsiveness that could be strengthened through sustained or personalized incentive structures. Conversely, Low-Exposure Cautious Drivers remain largely unaffected, suggesting that future programs could focus resources on driver segments with greater potential for change.

## 4. Discussion

The findings of this study provide empirical evidence that incentive-based telematics interventions can lead to measurable improvements in driving behavior, particularly among drivers exhibiting moderate risk profiles. Consistent with previous research highlighting the positive impact of feedback and gamification on driving performance [27,28], the results demonstrated that Balanced/Average Drivers significantly reduced both the frequency and intensity of speeding during the challenge periods. These findings suggest that individuals with moderate exposure and risk levels are more responsive to feedback and competition-based incentives, likely due to a combination of awareness, self-regulation capacity, and motivation for improvement. Similar behavioral responsiveness has been observed in other field trials of feedback and reward mechanisms, emphasizing the potential of such schemes to promote safer driving through behavioral nudges rather than punitive measures [10,29].

In contrast, High-Risk Drivers showed only partial but meaningful improvements, mainly in speeding intensity. While they responded positively to repeated challenges, their residual high levels of risky behavior suggest that a short-term, low-magnitude incentive may not be sufficient to modify deeply ingrained habits. This finding aligns with previous studies that have identified high-risk drivers as less sensitive to feedback-only or short-term interventions [30]. It indicates the need for more personalized, continuous, and higher-stakes incentive designs that combine feedback with coaching or adaptive goal-setting mechanisms. Moreover, the persistence of elevated mobile phone use among this group supports the idea that distraction-related behaviors may require distinct strategies, such as real-time deterrent feedback or driver assistance technologies [31].

The Low-Exposure Cautious Drivers, on the other hand, exhibited stable behavior throughout the study, with no statistically significant differences observed during the challenges. This stability is in line with their inherently safe baseline driving style and low driving exposure. Comparable results were reported by Mantouka et al. [17], where low-risk drivers demonstrated minimal room for improvement due to already safe performance levels. This finding underscores the importance of tailoring behavioral interventions to target driver segments with higher potential for behavioral change, ensuring the efficient allocation of resources in large-scale telematics or insurance applications.

From a broader perspective, the study reinforces the value of combining clustering analysis with telematics data to capture driver heterogeneity in real-world conditions. Previous research has successfully demonstrated that unsupervised learning can identify distinct behavioral typologies—ranging from cautious to aggressive drivers—and link these patterns to crash risk and insurance modeling [32,33]. The present study builds on this foundation by extending clustering analysis to evaluate behavioral responsiveness under incentive schemes, a relatively underexplored dimension in the literature. By identifying which driver subgroups are most receptive to feedback and incentives, telematics providers and policy-makers can design adaptive frameworks that personalize challenges, rewards, and feedback intensity according to driver risk level.

Beyond the above findings, the results of this study also have important implications for professional driver groups who are disproportionately exposed to road risk. Numerous studies have shown that taxi drivers, app-based ride-hailing workers, food-delivery couriers, and heavy-truck operators face elevated crash risk due to time pressure, algorithmic deadlines, and intensive smartphone interaction—behaviors that closely correspond to the speeding, harsh events, and distraction indicators examined in our analysis [34,35,36,37]. The observed responsiveness of the Balanced/Average and High-Risk drivers in our sample—particularly the significant reductions in speeding during both challenges—suggests that telematics-based, gamified incentive schemes could be a promising safety intervention for high-exposure professional drivers. For instance, delivery motorcyclists have been shown to increase risky maneuvers under time-sensitive incentives [34], while research on trucking indicates that heavy-vehicle operators are susceptible to severe speeding-related incidents [37]. Integrating incentive-based telematics into fleet management or platform governance frameworks could therefore support safer driving among these groups by providing continuous feedback, real-time risk awareness, and reward structures that counterbalance occupational time pressure.

Despite its promising findings, the study has some limitations. The pilot nature of the study inherently limited the participant pool to 86 active drivers. Non-parametric methods were applied to ensure valid statistical comparisons despite sample constraints. The relatively small sample size is acknowledged as a limitation, and future studies should aim for larger-scale replications to strengthen the statistical reliability and external validity of the results. Furthermore, the limited duration of the two incentive periods might have been insufficient to foster long-term behavioral change, especially for high-risk drivers who exhibited only minor improvements. The methodology developed in this study can be integrated into usage-based insurance schemes and corporate fleet safety management systems to promote safer and more efficient driving behavior. Scaling up this approach could contribute to broader adoption of evidence-based safety strategies across industries. Furthermore, external factors such as weather, traffic, or personal circumstances were not controlled for, which may influence driving behavior growth independently of the challenge.

In addition, because the study employed a pre–post comparison without a control group, the observed changes may partly reflect seasonal variation, weather conditions, shifts in traffic mix, or regression-to-the-mean. Incorporating additional contextual factors (e.g., route types, traffic density, weather conditions) [38,39] could refine cluster accuracy and help personalize interventions more effectively, while implementing interrupted time-series or mixed-effects models at the trip or week level could incorporate driver random intercepts and adjust for contextual factors.

Finally, a potential self-selection bias may exist, as participation in the study was voluntary. While this approach is ethically appropriate and widely used in behavioral research, it may introduce selection bias, as such participants often already exhibit higher safety awareness or greater technological familiarity compared to the broader driving population [40]. Addressing this issue in future work could enhance experimental design and interpretation by providing a more complete understanding of how gamification and incentive-based feedback influence a wider and more diverse group of drivers. In that context, Future research should apply intent-to-treat approaches or sensitivity analyses to assess how partial participation affects observed behavioral outcomes.

## 5. Conclusions

This research successfully demonstrated the potential of clustering techniques for profiling drivers using high-resolution telematics data. By categorizing participants into three profiles based on driving exposure and risk-related behaviors, the evaluation of behavioral changes was tailored during two distinguished competitive driving challenges. Overall, the results highlight the potential of integrating behavioral analytics, gamification, and telematics into scalable road safety solutions. Smartphone-based systems, such as the one deployed in this study, offer a cost-effective platform for continuous monitoring, feedback, and motivation, complementing traditional enforcement and education strategies. However, long-term evaluations are necessary to determine the sustainability of behavioral changes once incentives are removed.

## Figures and Tables

**Table 1:** Description of Challenges criteria and information.

No	Description—Criteria	Start Date	Finish Date	Gift
1	10 trips, 50 km, >80/100 speeding	5 December 2024	27 December 2024	2 € coupons
2	5 trips, 40 km, >75/100 overall score	30 January 2025	10 February 2025	5 € coupons

**Table 2:** Description of the variables used in the analysis.

Variable	Description
Total Trip Duration [s]	Total trip duration [sec]
Total Trip Distance [km]	Total trip distance [km]
Harsh accelerations [count]	Harsh acceleration events per trip [count]
Harsh braking [count]	Harsh braking events per trip [count]
Speeding Percentage [%]	Share of time over the speed limit per trip [%]
Speeding kmh_avg [km/h]	Average speed above the speed limits [km/h]
Mobile Use Percentage [%]	Share of mobile use per trip [%]
Harsh braking [count]	Harsh braking events per trip [count]

**Table 3:** Centroid centers and types for driver clusters.

Cluster	Distance Total	Duration Total	Harsh Accel.	Harsh Braking	Speeding Percent.	Speeding kmh_avg	Mobile Use Perc.	No of Drivers
Low-Exposure Cautious Drivers	432.41	46,955.90	0.38	0.60	2.23	2.03	3.68	42
Balanced/Average Drivers	1348.73	128,847.82	0.64	1.15	4.81	3.84	2.90	30
High-Risk Drivers	1536.63	138,875.29	2.65	2.33	10.37	6.30	7.24	14

**Table 4:** Mean values and Wilcoxon Test results for driving behavior indicators before and during the 1st challenge.

Profile	Variable	Mean_Before	Mean_During	*p*_Value
Low-Exposure Cautious Drivers	harsh_acc	0.42	0.39	0.12
Balanced/Average Drivers	harsh_acc	0.61	0.58	0.18
High-Risk Drivers	harsh_acc	2.41	2.31	0.26
Low-Exposure Cautious Drivers	harsh_brk	0.69	0.72	0.45
Balanced/Average Drivers	harsh_brk	1.14	1.09	0.52
High-Risk Drivers	harsh_brk	2.07	2.01	0.39
Low-Exposure Cautious Drivers	speeding_percentage	2.3	2.2	0.31
Balanced/Average Drivers	speeding_percentage	4.6	4.4	0.04
High-Risk Drivers	speeding_percentage	10.2	9.6	0.22
Low-Exposure Cautious Drivers	speeding_kmh_avg	2.1	1.9	0.34
Balanced/Average Drivers	speeding_kmh_avg	3.8	3.5	0.03
High-Risk Drivers	speeding_kmh_avg	6.1	5.7	0.03
Low-Exposure Cautious Drivers	mbu_percentage	3.4	3.7	0.17
Balanced/Average Drivers	mbu_percentage	3	3.1	0.25
High-Risk Drivers	mbu_percentage	7.2	6.5	0.14

**Table 5:** Mean values and Wilcoxon Test results for driving behavior indicators before and during the 2nd challenge.

Profile	Variable	Mean_Before	Mean_During	*p*_Value
Low-Exposure Cautious Drivers	harsh_acc	0.36	0.42	0.31
Balanced/Average Drivers	harsh_acc	0.66	0.70	0.38
High-Risk Drivers	harsh_acc	2.81	2.36	0.14
Low-Exposure Cautious Drivers	harsh_brk	0.50	0.56	0.16
Balanced/Average Drivers	harsh_brk	1.14	1.03	0.14
High-Risk Drivers	harsh_brk	2.33	1.94	0.35
Low-Exposure Cautious Drivers	speeding_percentage	2.13	2.46	0.35
Balanced/Average Drivers	speeding_percentage	4.80	3.67	0.01
High-Risk Drivers	speeding_percentage	10.66	8.47	0.02
Low-Exposure Cautious Drivers	speeding_kmh_avg	2.03	2.38	0.20
Balanced/Average Drivers	speeding_kmh_avg	3.84	3.08	0.01
High-Risk Drivers	speeding_kmh_avg	6.40	5.33	0.05
Low-Exposure Cautious Drivers	mbu_percentage	3.89	4.18	0.07
Balanced/Average Drivers	mbu_percentage	2.90	2.46	0.05
High-Risk Drivers	mbu_percentage	7.25	7.74	0.76

## Data Availability

The data presented in this study are available on request from the corresponding author.
